# Agreement and reliability statistics for shapes

**DOI:** 10.1371/journal.pone.0202087

**Published:** 2018-08-23

**Authors:** Travis B. Smith, Ning Smith

**Affiliations:** 1 Casey Eye Institute, Oregon Health & Science University, Portland, Oregon, United States of America; 2 Center for Health Research, Kaiser Permanente Northwest, Portland, Oregon, United States of America; University of New South Wales, AUSTRALIA

## Abstract

We describe a methodology for assessing agreement and reliability among a set of shapes. Motivated by recent studies of the reliability of manually segmented medical images, we focus on shapes composed of rasterized, binary-valued data representing closed geometric regions of interest. The methodology naturally generalizes to N dimensions and other data types, though. We formulate the shape variance, shape correlation and shape intraclass correlation coefficient (ICC) in terms of a simple distance metric, the Manhattan norm, which quantifies the absolute difference between any two shapes. We demonstrate applications of this methodology by working through example shape variance calculations in 1-D, for the analysis of overlapping line segments, and 2-D, for the analysis of overlapping regions. We also report the results of a simulated reliability analysis of manually delineated shape boundaries, and we compare the shape ICC with the more conventional and commonly used area ICC. The proposed shape-sensitive methodology captures all of the variation in the shape measurements, and it provides a more accurate estimate of the measurement reliability than an analysis of only the measured areas.

## Introduction

The reliability of an endpoint or outcome measure often must be estimated, for example in order to design and power a clinical trial appropriately. Several recently published studies in the field of ophthalmic imaging have attempted to assess the reliability of an anatomical endpoint whose measurement requires manually identifying the boundary of a retinal structure in a fundus image [1–3]. These studies assessed the reliability of the size—either the area or the meridional width—of the boundaries manually drawn by a group of raters. They have shown that the intra-rater repeatability and inter-rater reproducibility [4, 5] appear to be excellent, and reported an intraclass correlation coefficient as large as 0.996 [1].

These studies could be overly optimistic in their reliability assessments. The area or width is a simple summary value that discards most of the spatial and shape information contained within the manually drawn boundaries. Consequently, we expect the area or width would have less variation than the originally measured shapes from which they are created. Due to the geometric ambiguity that more than one shape can have the same area (or more than one line segment can have the same width), reliability assessments of the area (or width) could be biased toward better apparent performance than would actually be borne out if the complete measurement data were used instead. Because the true measurement here is the shape as defined by the manually delineated boundary, any agreement or reliability study should focus on shape differences rather than differences in their corresponding areas or widths.

Motivated by these observations and the need for a shape-sensitive approach, we have developed a methodology to assess the agreement and reliability [6] of shapes. While there have been numerous metrics developed to quantize the difference between two shapes [7, 8], there are comparatively fewer methods available to assess the variation or agreement among a group of shapes [9]. Because we consider the shapes to be measurements, we are interested in their absolute agreement; thus, we purposefully avoid any kind of difference mitigation or shape alignment, as is done with Procrustes analysis [7], which would artificially make the measurements more similar.

The primary component of our methodology is the shape variance, which is based on a shape distance metric that quantizes the total absolute difference between two shapes. Other statistics such as the shape correlation and shape ICC are then derived from the shape variance. We describe and formulate these shape statistics, and then present several examples to illustrate their application.

## Shape statistics

**Fig 1:**
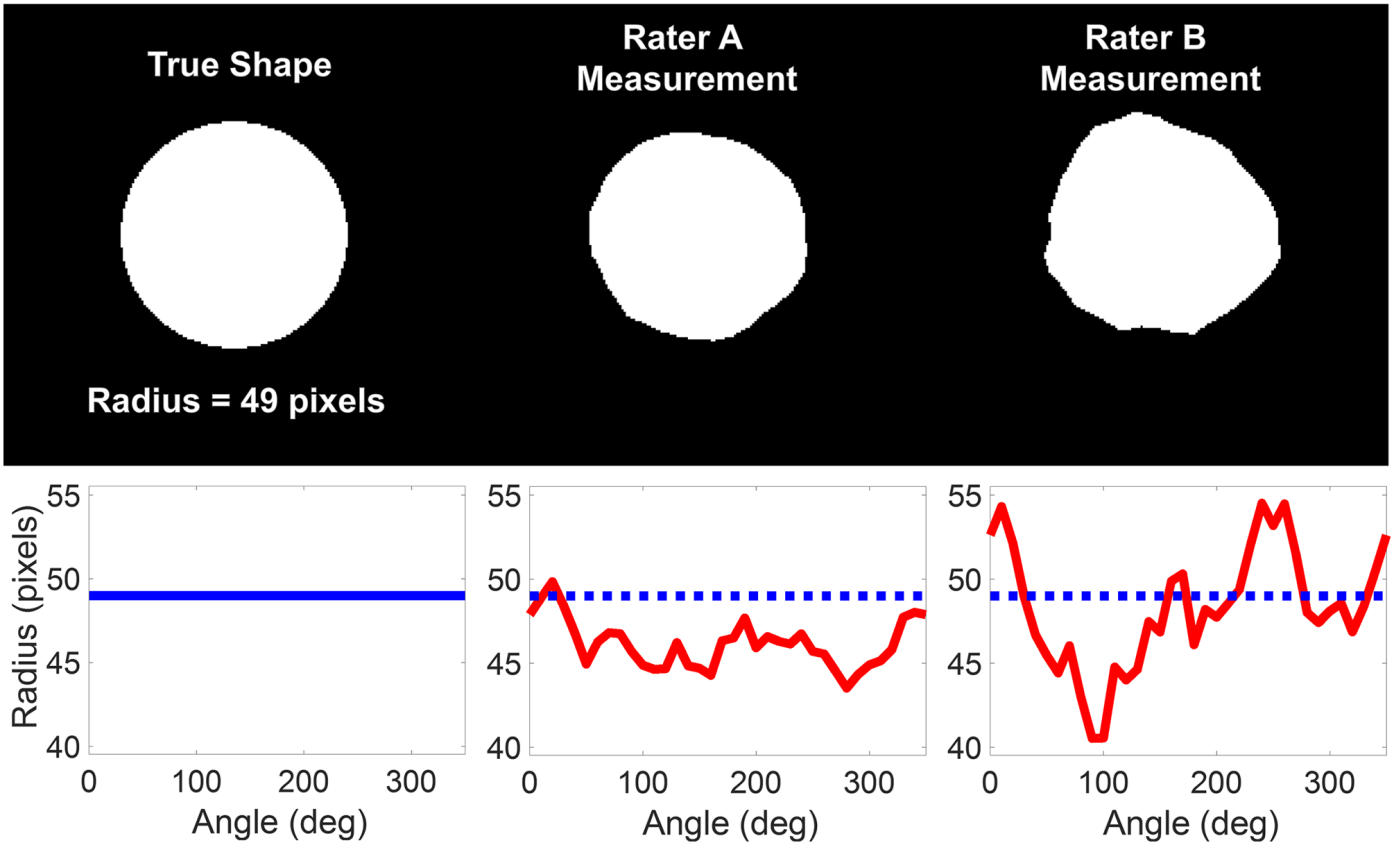
Example shapes from the simulated reliability study. From left to right, the top row depicts a 49-pixel radius circle representing the true shape, the simulated shape measurement for rater A, and the simulated measurement for rater B. The bottom row shows plots of the polar coordinate locations of the boundaries for the three shapes above. The boundaries for rater A and B were generated with zero-sum random walks to simulate measurement errors, with rater B having twice the error as rater A in delineating the shape boundary.

In this work, a shape is defined as an indicator function for some region or binary pattern of interest. The shape has value 1 only within the region, and 0 otherwise. For example, a shape could be a binary silhouette identifying the location of an anatomic structure of interest in a medical image, or a binary detection map indicating the positions of detected targets. Here, we focus on rasterized shapes that represent closed, solid geometric regions of interest such as those shown in Fig 1.

### Shape variance

The classical formulation for the variance of a set of *N* scalar-valued observations ${X=\{x_{i}\}}_{i=1}^{N}$ can be expressed as

$$\sigma^{2}=VAR(X)=\frac{1}{N}\sum_{i=1}^{N}{d(x_{i},\mu_{X})}^{2},$$ (1)

where *d*(*x*_*i*_, *μ*_*X*_) = |*x*_*i*_ − *μ*_*X*_| is a metric that quantifies the distance between any two values and *μ*_*X*_ is the sample mean.

In a similar fashion, for a set of shapes ${S=\{s_{i}\}}_{i=1}^{N}$, the shape variance is

$$\sigma_{s}^{2}=VAR(S)=\frac{1}{N}\sum_{i=1}^{N}{d(s_{i},\mu_{S})}^{2},$$ (2)

with the mean shape defined as the average across all shapes in the set,

$$\mu_{S}=\frac{1}{N}\sum_{i=1}^{N}s_{i},$$ (3)

which may not be binary valued. If the set consists of *m* × *n* rasterized shapes, then $s_{i}\in B^{m\times n}$ and the *m* × *n* mean shape is formed from the pixel-wise average.

The shape distance *d* in Eq (2) is a metric function that quantifies the difference between any two shapes as a scalar value, and is defined as the *ℓ*_1_ or Manhattan norm of the shape difference,

$$d(a,b)={\left\|a-b\right\|}_{1}.$$ (4)

For *m* × *n* rasterized shapes,

$$d(a,b)=\sum_{k=1}^{mn}\left|a[k]-b[k]\right|,$$ (5)

which is the total absolute pixelwise difference between the shapes. This function, $d:\;R^{m\times n}\times R^{m\times n}\to R_{\geq 0},$ provides a mapping from the multi-dimensional shape space to a scalar non-negative distance that represents the total absolute shape disagreement, and provides the same functionality as the distance in the classical variance formulation in Eq (1). Quantizing shape agreement with this function enables subsequent numerical and statistical analysis of the agreement among a set of shapes.

If shapes *a* and *b* are both binary valued, the shape distance *d*(*a*, *b*) is equivalent to the area of symmetric difference (ASD). The ASD between two binary shapes is the area of their union minus the area of their intersection [8, 10], or the area supported by one and only one of the two shapes. The ASD is equivalent to both the Hamming distance [11, 12] between the binary images and the area of the exclusive disjunction (XOR) of the binary images.

The ASD is related to the Jaccard distance [7, 9] between two shapes, which is one minus the ratio of their intersection to their union. We chose to use the ASD for the shape variance for several reasons. The ASD is more suitable for a shape variance definition because it more easily allows comparisons between binary shapes and their potentially non-binary means. The ASD generalizes straightforwardly as shown in Eq (4) to accommodate any continuous valued data type, whereas the Jaccard distance does not. Also, the ASD has units in the native space of the data, for example square pixels in the case of images, and retains the magnitude of the shape difference, thus making it more intuitive and interpretable than the Jaccard distance, which is a normalized measure having a magnitude of at most one.

The shape standard deviation *σ*_*s*_ can supplant conventional standard deviation to create statistical agreement metrics for shapes. For example, for a set of shapes, the repeatability coefficient (RC), which is the upper bound of the difference between any two shapes with 95% probability [13], and the Bland-Altman limits of agreement [14, 15] for the shapes can be found by simply substituting *σ*_*s*_ into their formulations. Another example is the shape covariance of two sets of shapes ${S=\{s_{i}\}}_{i=1}^{N}$ and ${T=\{t_{i}\}}_{i=1}^{N}$,

$$COV(S,T)=\frac{1}{N}\sum_{i=1}^{N}d(s_{i},\mu_{S})d(t_{i},\mu_{t}).$$ (6)

### Shape correlation coefficient and shape coefficient of determination

The Pearson correlation coefficient for two sets of shapes, *S* and *T* as defined above, is

$$\rho =\frac{COV(S,T)}{\sqrt{\sigma_{S}^{2}\sigma_{T}^{2}}}=\frac{\sum_{i=1}^{N}d(s_{i},\mu_{S})d(t_{i},\mu_{t})}{\sqrt{\sum_{i=1}^{N}d{\left(s_{i},\mu_{S}\right)}^{2}\sum_{i=1}^{N}{d(t_{i},\mu_{t})}^{2}}}.$$ (7)

Confidence intervals and p-values for *ρ* can be computed in the traditional manner. If we consider the shapes in set *T* to be the modeled or predicted shapes for those in set *S*, then the coefficient of determination is

$$R^{2}=1-\frac{\sum_{i=1}^{N}{d(s_{i},t_{i})}^{2}}{N\sigma_{s}^{2}}.$$ (8)

### Shape intraclass correlation coefficient

The shape intraclass correlation coefficient (ICC) is formulated by inserting the definitions for shape variance in Eq (2) and mean shape in Eq (3) into an analysis of variance (ANOVA) model. There are several different types of ICC available depending on the underlying model and experimental methodology [16], and all can be adapted to accommodate shapes. Here, we discuss one commonly used type based on a two-way, fully crossed random effects model. This type of ICC is appropriate to describe the absolute agreement among shape measurements from a group of *k* raters, randomly selected from the population of all raters, made on a set of *n* items. For example, the items could be medical images from a patient cohort. This ICC was given the label ICC(2,1) by Shrout and Fleiss [17] and the label ICC(A,1) by McGraw and Wong [18].

Let *x*_*ij*_ be the measured shape for the *i*^*th*^ item by the *j*^*th*^ rater, which can be considered the element at row *i* and column *j* in an *n* × *k* array of shapes. The between-row or between-item mean square is

$$MS_{R}=\frac{k}{n-1}\;\sum_{i=1}^{n}{d(\mu_{i},\mu )}^{2},$$ (9)

the between-column or between-rater mean square is

$$MS_{C}=\frac{n}{k-1}\;\sum_{i=1}^{n}{d(\mu_{j},\mu )}^{2},$$ (10)

and the residual mean square is

$$MS_{E}=\frac{SS-(n-1)MS_{R}-(k-1)MS_{C}}{\left(n-1)(k-1\right)},$$ (11)

with

$$\mu_{i}=\frac{1}{k}\;\sum_{j=1}^{k}x_{ij},$$

$$\mu_{j}=\frac{1}{n}\;\sum_{i=1}^{n}x_{ij},$$ (12)

$$\mu =\frac{1}{nk}\;\sum_{i=1}^{n}\sum_{j=1}^{k}x_{ij},$$

and

$$SS=\sum_{i=1}^{n}\sum_{j=1}^{k}d{\left(x_{ij},\mu \right)}^{2}.$$

Finally, the ICC is

$$ICC(2,1)=\frac{MS_{R}-MS_{E}}{MS_{R}+(k-1)MS_{E}+\frac{k}{n}(MS_{C}-MS_{E})}.$$ (13)

The F-statistic and confidence limits for the ICC can be calculated in the conventional manner [17, 19].

## Application examples

We provide analytical shape variance calculations for two example sets of shapes and compare the results with the classical variance of the shape sizes. The first example concerns 2-D shapes and their areas, and the second example focuses on 1-D shapes (line segments) and their widths. We also present a simulated reliability study to illustrate the advantages of assessing measurement repeatability using the shape ICC over the conventional ICC.

### Example 1: Circles with random radii

First, we compare the shape and area variances for a set of circles with random areas. Assume we have a set of *N* circles all centered at the origin, each with radius *r*_*i*_, where *r*_*i*_ is uniformly distributed between 0 and *r*_*max*_. The binary-valued *i*^*th*^ circle is

$$c_{i}\left(r\right)=\{\begin{array}{c} 1,\;r\leq r_{i}\\ 0,\;r>\;r_{i} \end{array}.$$ (14)

The mean shape is the circularly symmetric function

$$m(r)=E[\frac{1}{N}\sum_{i=1}^{N}c_{i}(r)]=E[c_{i}(r)]=\frac{r_{max}-r}{r_{max}},$$ (15)

which is a cone whose height decreases linearly from 1 to 0 as *r* increases from 0 to *r*_*max*_.

The difference between circle *c*_*i*_ and the mean *m* is

$$f_{i}\left(r\right)=c_{i}\left(r\right)-m\left(r\right)=\{\begin{array}{rr} \frac{r}{r_{max}}, & 0\leq r\leq r_{i}\\ \frac{r-r_{max}}{r_{max}}, & r_{i}<r\leq r_{max} \end{array}.$$ (16)

From Eq (4), the shape distance between circle *c*_*i*_ and the mean *m* is the scalar value

$$d_{i}={\left|f_{i}(r)\right|}_{1}=\int_{0}^{2\pi }\int_{0}^{r_{max}}|f_{i}(r)|rdrd\theta =\frac{\pi }{3}(\frac{4r_{i}^{3}}{r_{max}}-3r_{i}^{2}+r_{max}^{2}),$$ (17)

the square of which is

$$d_{i}^{2}=\frac{\pi^{2}}{9}(\frac{16r_{i}^{6}}{r_{max}^{2}}-\frac{24r_{i}^{5}}{r_{max}}+9r_{i}^{4}+{8r_{i}^{3}r}_{max}-6r_{i}^{2}r_{max}^{2}+r_{max}^{4}).$$ (18)

Because *r*_*i*_~*U*(0, *r*_*max*_), the *n*^*th*^ moment of *r*_*i*_ is $E[r_{i}^{n}]=r_{max}^{n}/\left(n+1\right)$. Therefore, the expected shape variance is

$$E[\sigma_{s}^{2}]=\frac{1}{N}\sum_{i=1}^{N}E[d_{i}^{2}]=\frac{{38\pi }^{2}}{315}r_{max}^{4}.$$ (19)

In comparison to the shape variance, the expected variance of the areas corresponding to the shapes is

$$E[\sigma^{2}]=E[a_{i}^{2}]-E{\left[a_{i}\right]}^{2}=\frac{{4\pi }^{2}}{45}r_{max}^{4},$$ (20)

where $a_{i}=\pi r_{i}^{2}$ is the area of the *i*^*th*^ circle. Thus, the shape variance is 19/14 or 36% larger than the area variance.

This example shows that when shapes differ only in their area or size and not in their position or boundary pattern, then the shape variance is equivalent to the area variance to within a scale factor. Thus, the two types of variance convey the same information, as expected. The scale factor will not affect the ICC and other statistics that are based on a ratio of variances.

### Example 2: Lines with random locations

Next, we compare the shape and width variances for a set of line segments with random positions. Each of *N* lines has the same width *w* but a normally distributed center point *x*_*i*_, with $x_{i}\sim N(0,\sigma_{x}^{2})$. The binary-valued *i*^*th*^ line is

$$l_{i}\left(x\right)=\{\begin{array}{c} 1,\;\left|x-x_{i}\right|\leq \frac{w}{2}\\ 0,\;\left|x-x_{i}\right|>\frac{w}{2} \end{array}.$$ (21)

Following the same sequence of equations as in Example 1,

$$m(x)=E[l_{i}(x)]=\frac{w}{\sqrt{2\pi \sigma_{x}^{2}}}\;e^{\frac{-x^{2}}{2\sigma_{x}^{2}}},$$ (22)

$$f_{i}\left(x\right)=l_{i}\left(x\right)-m\left(x\right)=\{\begin{array}{rr} 1-m\left(x\right), & \left|x-x_{i}\right|\leq \frac{w}{2}\\ -m\left(x\right), & \left|x-x_{i}\right|>\frac{w}{2} \end{array},$$ (23)

$$d_{i}={\left|f_{i}(x)\right|}_{1}=\int_{-\infty }^{\infty }|f_{i}(x)|dx\approx 2w(1-m(x_{i})),$$ (24)

$$d_{i}^{2}\approx 4w^{2}-8w^{2}m(x_{i})+4w^{2}{m(x_{i})}^{2},$$ (25)

$$E[\sigma_{s}^{2}]=\frac{1}{N}\sum_{i=1}^{N}E[d_{i}^{2}]\approx 4w^{2}-\frac{{4w}^{3}}{\sqrt{\pi }\sigma_{x}}+\frac{2w^{4}}{\sqrt{3}\pi \sigma_{x}^{2}}.$$ (26)

For Eq (24), we have assumed that *w* ≪ *σ*_*x*_ so that the interval [*x*_*i*_ − *w*/2, *x*_*i*_ + *w*/2] is small enough that *m*(*x*) ≈ *m*(*x*_*i*_).

In comparison to the shape variance, the expected variance of the widths of the shapes is

$$E[\sigma^{2}]=0,$$ (27)

because all of the lines have the same width.

This example shows that when shapes differ only in their position but are otherwise identical, the shape variance captures these differences whereas a conventional variance based on the shape size does not. Here, the shape variance is proportional to both the position variation *σ*_*x*_ and the line width *w*.

### Example 3: ICC of manually marked boundaries

Finally, we compare the conventional area ICC and shape ICC in a simulated reliability study. This study mimics the type described in the Introduction in which several human raters delineate an anatomical structure in medical images acquired from a cohort of patients. In such a study, each rater inspects the image from each patient and outlines the structure of interest. The structure’s area is the endpoint of interest, and the inter-rater reliability of the measurements is being determined. The conventional agreement statistic is the ICC of the measured areas. We compare this with the shape ICC, which is created directly from the raters’ shape measurements and therefore captures all of the measurement variation and provides a more accurate reliability assessment. For both conventional and shape ICC, we use type ICC(2,1) [17], as formulated in Eq (13).

In our simulated study, there were 100 patients and 2 raters. The images were 201x201 pixels, and the anatomy of interest for each patient was represented by a circle with a radius uniformly distributed between 0 and *r*_*max*_ = 50 pixels. The measurement error for outlining each shape was generated in polar coordinates and represented by a radially oriented deviation from the true boundary. Each deviation was generated from a 1D random walk over the 2*π* radians around the circle perimeter, with underlying step sizes that were normally distributed with mean zero and standard deviation *σ*_*e*_. The walks were zero-sum to ensure that the start (0 radians) and end (2*π* radians) of each deviation were identical, so that the rater’s measured outline did not contain unrealistic discontinuities. Measurement errors for rater A and rater B were set to *σ*_*e*,*A*_ = 1 pixel and *σ*_*e*,*B*_ = 2 pixels, respectively. Example shapes from this simulated study are shown in Fig 1.

The average results from 40 repetitions of this study with the above parameters are as follows. The average measured area of all shapes was 2612 square pixels for rater A, and 2676 square pixels for rater B. Compared to the expected mean shape area of $E[\pi r_{i}^{2}]=\frac{\pi }{3}r_{max}^{2}=2618$, rater A with the smaller *σ*_*e*,*A*_ was closer on average. The average measured area for rater B was larger because of a positive bias in the simulated deviations that was more pronounced with larger *σ*_*e*_, especially for smaller shapes. This positive bias occurred because simulated deviations on the inner side of the true boundary—where the rater’s measurement was approaching the origin—were rounded off to avoid exceeding the circle radius, thereby imparting a floor effect that limited the deviation magnitude, skewed the measurements outward from the boundary, and led to larger measured areas.

The ICC of the measured areas was 0.94 (95% CI: 0.92–0.96), which appears to show good reliability. However, the ICC of the measured shapes was 0.78 (95% CI: 0.69–0.85), significantly smaller (P < 0.001) than the area ICC. This reduction in ICC reflects the additional between-rater variation captured by the shape-sensitive approach that was missed by the area-only analysis. This example demonstrates the importance of incorporating shape into reliability studies of summary measures such as the area or width of geometric regions. The code and data to reproduce these ICC values are available in S1 and S2 Files.

**Fig 2:**
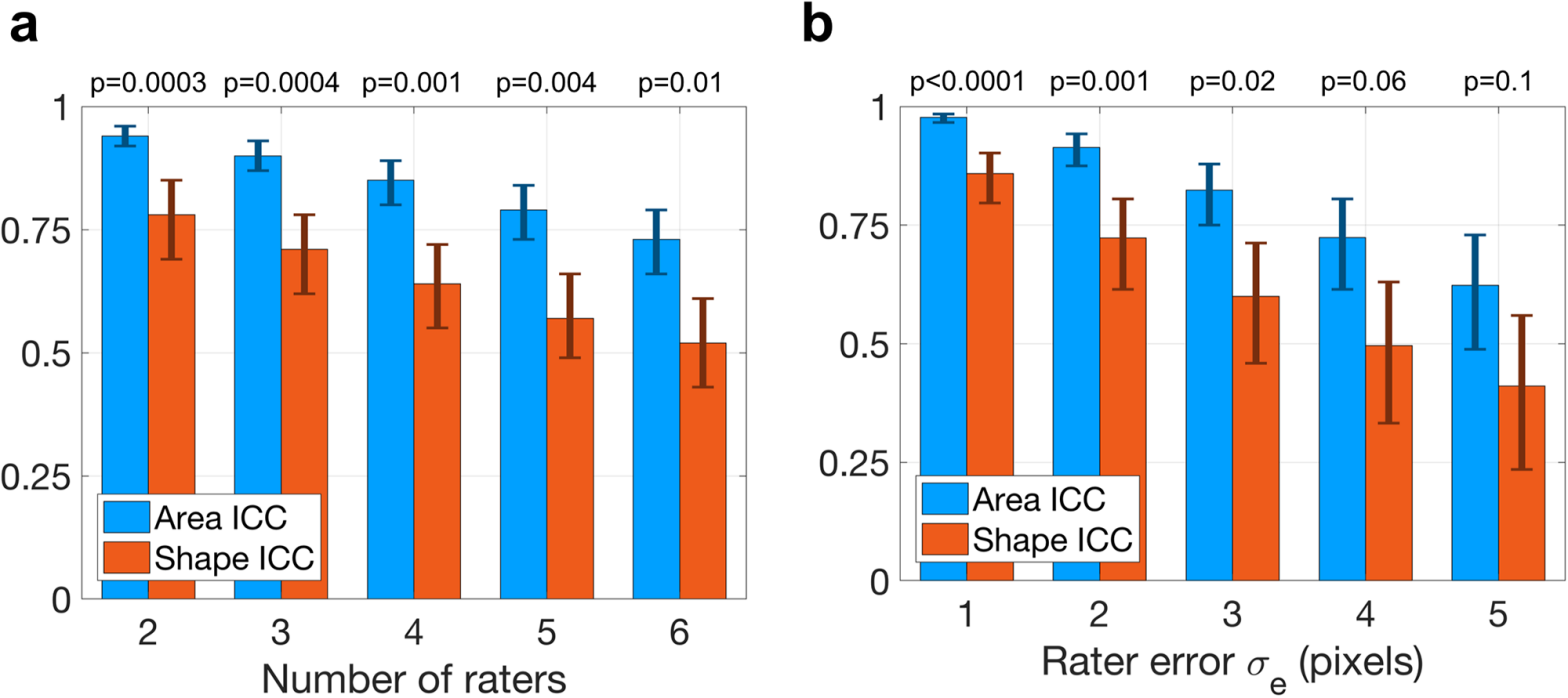
ICC trends from simulated reliability studies. (a) The shape and area ICC are reported from simulated studies with increasing numbers of raters in which additional raters had larger measurement errors. The error bars represent the 95% confidence intervals, and the p-values are shown for the difference between shape and area ICC. (b) The shape and area ICC are reported as rater error increases for two raters with identical measurement error standard deviation *σ*_*e*_.

To better understand the relationships between ICC and rater inaccuracy in this example, we extended the simulation to include more raters and larger measurement error. Fig 2a shows the area and shape ICC from studies simulated as described above but with additional raters, where in each study the *i*^*th*^ rater has error *σ*_*e*,*i*_ = *i* pixels. For example, with four raters, the rater measurement errors were 1, 2, 3, and 4 pixels. Fig 2b shows the ICC trends as measurement error *σ*_*e*_ increases in studies with two raters having equal error statistics. In both plots, the difference between shape ICC and area ICC becomes more significant as rater accuracy improves, indicating that the importance of shape information in such studies grows with the skill of the raters.

## Discussion

Studies of measurements of a region’s boundary should include shape-sensitive statistics in their analysis. The area and width are summary measures created by distilling the shape boundary to a simple scalar value, and they exhibit inherently less variation than the original shape boundary measurements which generated them. Shape ICC captures all of the measurement variation and works naturally and directly with the raw measurements. The shape ICC is a more accurate estimate of measurement reliability than the area ICC or width ICC. Reliability analyses that neglect this variation could yield a misleadingly large ICC.

A shape-sensitive framework offers an important additional benefit, as well. The ASD of binary shapes *a* and *b* is separable into two components: the area inside *a* but outside *b* (denoted *a*\*b*), and the area inside *b* but outside *a* (*b*\*a*). If, for example, shapes *a* and *b* represent anatomy before and after treatment, then the first component quantifies the reduction in size due to the treatment, and the second component quantifies the growth. Compared to a conventional difference of areas, this shape-sensitive approach provides additional information about the positive and negative components of the difference, which creates new opportunities for analyzing and understanding the data.

The methodology presented here is flexible and extensible. Although the focus of this work has been on binary images representing closed geometric regions, the methodology is applicable to the analysis of any kind of discretized binary-valued pattern and all possible 2^*mn*^ images within the domain $B^{m\times n}$. Furthermore, it generalizes straightforwardly to any type of continuous-valued data, not just binary data, for which basic arithmetic operations are defined. It also generalizes naturally to accommodate data of any dimension, making it useful to 3D imaging for example.

## Supporting information

S1 File Source code. MATLAB code to calculate shape ICC and area ICC, and a script that reproduces the results from Example 3, to be used with S2 File.
(PDF)

S2 File Complete data set. MATLAB .mat file containing all simulated shapes used in Example 3.
(ZIP)
