# Supplementary material for: Agreement and reliability statistics for shapes
Source: PLoS One. 2018 Aug 23;13(8):e0202087. doi: 10.1371/journal.pone.0202087 (PMC6107162; doi:10.1371/journal.pone.0202087)
Supplement: S1 File — MATLAB code to calculate shape ICC and area ICC, and a script that reproduces the results from Example 3, to be used with S2 File. (PDF) [file pone.0202087.s001.pdf]

shapeICC.m

```
function icc = shapeICC(M)
%SHAPEICC Shape intraclass correlation coefficient.
%   icc = shapeICC(M)
%
%   Calculates ICC is for a two-way, fully crossed random efects model.
%   This type of ICC is appropriate to describe the absolute agreement
%   among shape measurements from a group of k raters, randomly selected
%   from the population of all raters, made on a set of n items.
%   Shrout and Fleiss: ICC(2,1)
%   McGraw and Wong:   ICC(A,1)
%
%   M is the stack of N-dimensional rasterized shape measurements
%      The dimensions of M are Ny x Nx x ... x n x k, where
%         Ny x Nx x ... is the size of each N-dimensional rasterized shape
%         n is the # of subjects / groups
%         k is the # of raters
%
%      For example, with 2-D shapes,
%      M(:,:,2,3) would be the shape corresponding to subject 2, rater 3

% setup
sizeM = size(M);
k = sizeM(end);
n = sizeM(end-1);
Npix = prod(sizeM(1:end-2)); % Npix = Ny * Nx * ... = total # of pixels in
each shape

M = reshape(M,[Npix n k]); % vectorize each shape into an Npix-length column
array
M = single(M); % in case M is logical, need it to be single to do floating-
point ops on it

%%%%%%%%%%%%%%%%%%%%%%%%%%%%%%%%%%%

% means
u1 = squeeze(mean(M,2)); % Npix x k
u2 = mean(M,3); % Npix x n
u = mean(u1,2); % Npix x 1

%%%%%%%%%%%%%%%%%%%%%%%%%%%%%%%%%%%

SS = 0;
for ii=1:n
    for jj=1:k
        d = abs(M(:,ii,jj) - u);
        SS = SS + sum(d(:))^2;
    end
end

%%%%%%%%%%%%%%%%%%%%%%%%%%%%%%%%%%%

MSR = 0;
for ii=1:n
    d = abs(u2(:,ii) - u);
```

```
    MSR = MSR + sum(d(:))^2;
end
MSR = k/(n-1) * MSR;

%%%%%%%%%%%%%%%%%%%%%%%%%%%%%%%%%%%

MSC = 0;
for jj=1:k
    d = abs(u1(:,jj) - u);
    MSC = MSC + sum(d(:))^2;
end
MSC = n/(k-1) * MSC;

%%%%%%%%%%%%%%%%%%%%%%%%%%%%%%%%%%%

MSE = (SS - (n-1)*MSR - (k-1)*MSC) / ((n-1)*(k-1));
icc = (MSR - MSE) / (MSR + (k-1)*MSE + k/n*(MSC-MSE));

end
```

ICC.m

```
function icc = ICC(M)
%ICC Intraclass correlation coefficient.
%   icc = ICC(M)
%
%   Calculates ICC is for a two-way, fully crossed random efects model.
%   This type of ICC is appropriate to describe the absolute agreement
%   among shape measurements from a group of k raters, randomly selected
%   from the population of all raters, made on a set of n items.
%   Shrout and Fleiss: ICC(2,1)
%   McGraw and Wong:   ICC(A,1)
%
%   M is the array of measurements
%      The dimensions of M are n x k, where
%         n is the # of subjects / groups
%         k is the # of raters

[n, k] = size(M);

u1 = mean(M,1);
u2 = mean(M,2);
u = mean(M(:));

SS = sum((M(:)-u).^2);
MSR = k/(n-1) * sum((u2-u).^2);
MSC = n/(k-1) * sum((u1-u).^2);
MSE = (SS - (n-1)*MSR - (k-1)*MSC) / ((n-1)*(k-1));
icc = (MSR - MSE) / (MSR + (k-1)*MSE + k/n*(MSC-MSE));

end
```

go.m

```
% This script reproduces the shape ICC and area ICC results from Example 3

clear;

% setup
% the data array contains all the measured shapes from the simulated
experiments
load data.mat
[Ny,Nx,Npts,Nraters,Nreps] = size(data);
[shape_icc,area_icc] = deal(zeros(Nreps,1));

% calculate shape and area ICC for each repetition of the simulated
experiment
for rr=1:Nreps
    fprintf('Analyzing rep %d of %d\n',rr,Nreps);

    measured_shapes = data(:,:,:,:,rr);
    measured_areas = squeeze(sum(sum(measured_shapes,1),2)); % reduce the
shape to its area

    shape_icc(rr) = shapeICC(measured_shapes); % shape ICC for all shapes for
this rep
    area_icc(rr) = ICC(measured_areas); % area ICC for all shape areas for
this rep
end

fprintf('Mean area ICC:  %f\n',mean(area_icc));
fprintf('Mean shape ICC: %f\n',mean(shape_icc));

%%%%%%%%%%%%%%%%%%%%%%%%%%%%%%%%%%%%%%%

% show some of the measured shapes

pts = [25 50 75]; % which "patients" to show
raters = [1 2]; % which raters to show
rep = 7; % which rep to show
S = logical([]);

for pp=1:numel(pts)
    s = logical([]);
    for rr=1:numel(raters)
        s = [s data(:,:,pts(pp),rr,rep)];
    end
    S = [S; s];
end

figure; imshow(S);
```
